# Derivation and internal validation of an equation for albumin-adjusted calcium

**DOI:** 10.1186/1472-6890-8-12

**Published:** 2008-11-27

**Authors:** Matthew T James, Jianguo Zhang, Andrew W Lyon, Brenda R Hemmelgarn

**Affiliations:** 1 Department of Medicine, University of Calgary, Calgary, Alberta, Canada; 2 Department of Community Health Sciences, University of Calgary, Calgary, Alberta, Canada; 3 Calgary Laboratory Services, Calgary, Alberta, Canada

## Abstract

**Background:**

Previously published equations to adjust calcium for albumin concentration may vary depending on factors such as the type of reagents used. Albumin-adjusted calcium equations derived from laboratories using the bromocresol purple (BCP) albumin binding reagent have not been described.

**Methods:**

The linear regression equation for the binding of calcium and BCP-albumin was derived in a cohort of 4613 outpatients, and the albumin-adjusted calcium equation was internally validated in a separate cohort of 1538 subjects. The performance of this equation was compared with a previously published equation (*adjusted [Ca](mmol/L) = total [Ca](mmol/L) + 0.02 (40 - [albumin] (g/L)) *in 343 subjects with albumin < 33 g/L (below reference range).

**Results:**

The local adjustment equation was expressed by the relationship; *adjusted [Ca](mmol/L) = total [Ca](mmol/L) + 0.012 (39.9 - [albumin](g/L))*. The equation showed evidence of good internal validity (shrinkage value of adjusted r^2 ^= -0.0059). Classification of calcium status differed between the two equations in 47 of 343 subjects with low serum albumin (weighted κ = 0.46; moderate agreement).

**Conclusion:**

A locally derived and internally validated albumin-adjusted calcium equation differed from previously published equations and resulted in important differences in classification of calcium status in hypoalbuminemia patients. Individual laboratories should determine their own linear regression equation for calcium on albumin rather than relying on published formulas.

## Background

Measurement of serum total calcium is commonly used to assess calcium status. At physiological pH albumin binds approximately 45% of serum total calcium. Variation in serum albumin concentration therefore alters the concentration of serum total calcium, while the concentration of physiologically important ionized calcium remains constant [1]. Equations to adjust total calcium for albumin, such as the frequently cited '*adjusted [Ca](mmol/L) = total [Ca](mmol/L) + 0.02 (40 - [albumin](g/L))' *are routinely used in clinical practice to give an estimate of calcium concentration in patients with hypoalbuminemia [1-3]. These equations were derived in single laboratories over 20 years ago by determining the linear regression relationship of serum calcium on albumin concentration in patients deemed free of calcium disorders. Changes in laboratory analytic techniques, including a shift to the use of the bromocresol purple (BCP) albumin binding reagent, now used by approximately half of clinical laboratories that participate in College of American Pathologists proficiency testing surveys, may influence the serum total calcium to albumin relationship [4,5] and impact the assessment of calcium status when using these equations.

The purpose of this study was to derive and internally validate an albumin-adjusted calcium equation by applying linear regression to our own laboratory data for individual patient total calcium and serum albumin measurements. We hypothesized that 1) a locally derived equation would differ from the previously published equations, and 2) that there would be differences in the classification of calcium status between equations when applied to patients with hypoalbuminemia.

## Methods

Consecutive outpatients with simultaneous serum total calcium and albumin measurements determined by Calgary Laboratory Services (CLS) over a 2 month time period were included. CLS provides laboratory testing for the Calgary Health Region (population 1.1 million) using instruments and reagents from a single vendor (Roche Diagnostics Ltd, Laval, PQ, Canada). Tests were conducted using the Arsenazo III dye binding method for calcium and the BCP reagent for albumin. Quality was satisfactory during the study period and assured by use of multi-rule quality control procedures at three levels [6], maintenance of clinical laboratory accreditation by the College of Physicians and Surgeons of Alberta with successful evaluation by proficiency testing programs provided by the College of American Pathologists (Northfield, IL, USA) and Ceqal (Vancouver, Canada). Exclusion criteria were: age < 18 years, serum creatinine > 200 μ mol/L, albumin < 20 g/L or > 50 g/L, total calcium > 3.0 mmol/L, or elevations in serum parathyroid hormone, alkaline phosphatase, or alanine transaminase above the reference range (reflecting the presence of conditions which may influence serum calcium levels). Vitamin D concentrations were not assessed. Only the first simultaneous total calcium and albumin result per patient during the study period were employed. The distribution of serum calcium concentrations was assessed graphically and found to follow a normal distribution.

The study cohort was divided randomly into a 75% derivation sample and a 25% validation sample. A simple (ordinary least squares) linear regression equation for the association between total calcium and albumin was determined from the derivation sample [4,5,7,8], and the regression equation was then cross-validated in the validation sample in the following manner. First, the validity of the regression equation was examined by calculating the amount of shrinkage in the predictive power of the equation [9]. This was carried out by applying the regression equation derived from the derivation sample to the validation sample to obtain a predicted calcium value for each subject. Measured calcium was then regressed on predicted calcium to obtain an estimate of the variance accounted for in the validation sample. The adjusted r^2 ^for the validation sample was subtracted from that of the derivation sample to arrive at an estimate of the amount of shrinkage, an indication of how much the predictive ability decreases when the equation is applied to other samples. If the shrinkage is small the regression is considered internally valid [9]. Moreover, in a sensitivity analysis a bootstrapping procedure was undertaken as an additional assessment of the internal validity. The Bootstrapping procedure conceptually involves copying samples of a data set on top of themselves infinitely creating a mega data file [10]. A total of 1,000 re-samples were randomly drawn with replacement from the full sample. Analyses were then conducted on each new sample with regression parameters estimated for each sample including the original sample. Bootstrapping can provide regression coefficients, standard errors, and confidence intervals [10].

To compare discrepancies in the classification of calcium status with a published equation [1], corrected calcium concentrations were determined with each formula for 343 subjects with albumin concentration below the laboratory reference range (albumin < 33 g/L). The pattern of the individual differences for the hypoalbuminemic patients as well as the mean agreement with 95% limits of agreement was assessed using a Bland-Altman plot. This technique plots the difference in calcium concentration between the two equations against the mean of the two values for each subject [11]. Agreement in classification of calcium status between formulas was also assessed as follows; as hypo-, normo-, and hyper-calcemic by the weighted Kappa statistic [12], and as within or outside the laboratory reference range using McNemar's test. All analyses were conducted with the use of SAS software (version 8.01, SAS Institute Inc., Cary, NC, USA) or STATA (version 8.0, Stata Corp., College Station, TX, USA). The institutional review board at the University of Calgary approved the study.

## Results

**Figure 1:**
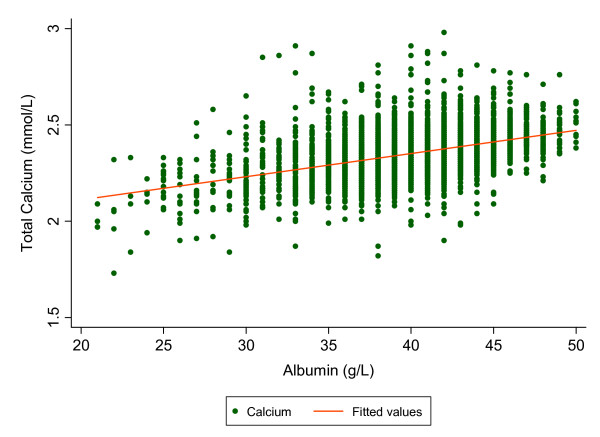
Linear regression relationship of serum total calcium on albumin derived from a cohort of 4613 patients with simultaneous total calcium and BCP-albumin measurements.

The cohort consisted of 6151 subjects, with 60% females, and a mean age of 55 years. The mean (SD) calcium and albumin concentrations were 2.35 (0.12) mmol/L and 39.9 (4.3) g/L respectively. The relationship between serum total calcium and albumin obtained from the derivation subset (n = 4613) is shown in Figure 1, and was expressed by the regression equation: *total [Ca](mmol/L) = 0.012 [albumin (glL)] + 1.871*, (correlation coefficient, r = 0.435). The working form of the equation was derived as outlined in Appendix 1. The resulting equation was expressed as: *adjusted [Ca](mmol/L) = total [Ca](mmol/L) + 0.012 (39.9 - [albumin](g/L))*. Evidence of good internal validity was confirmed by an adjusted r^2 ^shrinkage value of -0.0059 when the equation was applied to the validation subset (n = 1538). Similar results were obtained from the bootstrapping procedure, confirming the validity of the derived equation.

To assess agreement between the derived equation and the previously published equation [1] we compared adjusted-calcium results using the locally derived equation and the published equation to the 343 subjects with albumin < 33 g/L (below reference range). Within these subjects the median (interquartile range) for albumin was 30 (27 – 31) g/L, and the mean (standard deviation) for calcium was 2.36 (0.13) mmol/L using the locally derived forumula versus 2.45 (0.13) mmol/L using the published equation. The Bland-Altman plot produced a mean difference in adjusted calcium concentration between formulas of 0.09 mmol/L, 95% limits of agreement 0.043 to 0.136 (see Additional file 1). The resulting classification of calcium status with each equation is shown in Table 1. Classification of calcium status as hypocalcemic, normocalcemic, or hypercalcemic (according to the laboratory reference range) differed between the two equations in 47 of the 343 subjects. Five (1.5%) subjects were classified as hypocalcemic with the local equation but normocalcemic with the published formula. Forty two (12.2%) subjects were classified as normocalcemic with the local equation but hypercalcemic by the published formula. The weighted kappa statistic was κ = 0.46 (95% confidence interval 0.33 – 0.58), a value representing moderate agreement between the published and local equations. When calcium status was classified as within or outside the laboratory reference range, a significant p value of p < 0.0001 was obtained using the McNemar test for the difference between the two equations.

**Table 1:** Classification of 343 patients with hypoalbuminemia (albumin < 33 g/L) as hypo-, normo, or hyper-calcemic using local and previously published albumin-adjusted calcium equations.

	Local Equation*^1^		
	
Published Equation*^2^	**Hypocalcemia** (Ca < 2.10 mmol/L)	**Normocalcemia** (Ca 2.10 – 2.55 mmol/L)	**Hypercalcemia** (Ca > 2.55 mmol/L)
**Hypocalcemia** (Ca < 2.10 mmol/L)	2	0	0
**Normocalcemia** (Ca 2.10 – 2.55 mmol/L)	**5**	272	0
**Hypercalcemia** (Ca > 2.55 mmol/L)	0	**42**	22

*^1 ^adjusted [Ca](mmol/L) = total [Ca](mmol/L) + 0.012 (39.9 - [albumin](g/L))

*^2 ^adjusted [Ca](mmol/L) = total [Ca](mmol/L) + 0.02 (40 - [albumin](g/L))

## Discussion

We derived an albumin-adjusted calcium equation using local laboratory data that differs from those previously published. Its application in patients with abnormal low albumin concentration illustrates clinically important differences in the classification of calcium status compared with the most commonly used published adjustment equation. Our findings suggest that the use of a local equation for albumin adjusted-calcium in hypoalbuminemic patients would result in a small increase in the number of patients characterized as hypocalcemic and significantly fewer patients being characterized as hypercalcemic.

Measurement of total calcium concentration remains a common initial test in the assessment of calcium status. While accurate measurement of ionized calcium would be ideal, its strict sampling handling requirements make it more prone to measurement error and impractical to use as a screening test [4]. Thus remains the need to utilize serum total calcium, adjusted for albumin concentration, particularly in many outpatient settings. The clinical significance of a difference in the adjustment equation such as we observed in this study is dependent on the prevalence of abnormal albumin and calcium concentrations within the population studied [13]. Application in populations with a greater prevalence of hypoalbuminemia and calcium disorders would have a larger impact on classification.

While we did not seek to determine the reasons for the difference between our equation and other published versions, a likely explanation may be differences in analytical techniques employed. Ashby et al. noted a change in the regression coefficient for binding of calcium on albumin within a single laboratory following a change of analytical methodology [5]. Similarly, Barth et al. observed differences in this relationship between laboratories using similar assays [8]. Changes in the formulation of the albumin binding bromocresol green (BCG) reagent have been thought to be most responsible, mediated by differences in the degree to which the BCG reagent reacts with serum globulins [8]. In our study the BCP reagent was used for albumin measurement, while all previous reported adjustment equations were derived from work using the BCG reagent [1-5,8]. Systematic differences between the BCP and BCG reagents have been described; some authors have reported overestimation of albumin with BCG due to globulin binding, while others have shown greater variability in albumin levels with BCP [14,15]. Regardless of its relative advantages and shortcomings, the BCP method was used in over one-third of US laboratories surveyed in 1997 [14], and now by approximately half of clinical laboratories that participate in College of American Pathologists proficiency testing surveys. Our results raise concern about the validity of previous published equations for albumin-adjusted calcium when applied to calcium and albumin results obtained from these laboratories.

There are limitations to our study. First, we did not measure ionized calcium concentrations to determine the accuracy of the hypercalcemic, normocalcemic, and hypocalcemic classifications using different equations. Comparison against such a gold standard would be needed to confirm the greater validity of the locally derived equation. Second, the equation we report is applicable only over the albumin range studied (20–50 g/L), although this would include the majority of the population with low serum albumin levels to which this equation would be applied. Third, generalization of our equation beyond our health region may be limited given the interlaboratory variation previously described. Furthermore, generalization to inpatient settings may also be inappropriate since our equation was derived in an outpatient setting and previous literature has demonstrated poor reliability of this approach in the critically ill [16]. Fourth, the equation may not be valid in situations where the BCP method may underestimate albumin concentration, namely in the presence of hyperbilirubinemia or in patients receiving dialysis due to the presence of an endogenous ligand [14]. Finally, we did not compare the effects of measurement of serum albumin using different assays (ie. BCP versus BCG) when comparing adjusted calcium results. While we cannot be certain the use of albumin binding agent explains these effects, our findings more importantly illustrate the extent of differences that may exist between different settings.

## Conclusion

In conclusion, a locally derived and internally validated albumin-adjusted calcium equation differed from previously published equations and resulted in important differences in classification of calcium status compared to results from a previously published equation. Our results illustrate that clinicians should be cautious applying a previously published albumin-adjusted calcium equation in modern settings with different analytical techniques. Using the methods described in this report institutions can derive their own equations rather than relying upon existing published formulas.

## Abbreviations

Ca: Calcium; BCP: Bromocresol purple; BCG: Bromocresol green; CLS: Calgary Laboratory Services.

## Competing interests

The authors declare that they have no competing interests.

## Authors' contributions

MJ, AL, and BH contributed to study conception and design, acquisition and interpretation of data, and were involved in drafting the manuscript and revising it critically for important intellectual content. JZ contributed to analysis and interpretation of data. All authors gave final approval of the version to be published.

## Appendix: Derivation of working form of albumin-adjusted calcium equation

$$\begin{array}{c} Adjusted\left[Ca\right]=total\left[Ca\right]-\left(slope\times \left[albumin\right]\right)+\left(meanl\;total\left[Ca\right]-intercept\left[Ca\right]\right)\\ =total\left[Ca\right]-\left(0.012\times \left[albumin\right]\right)+\left(2.350-1.871\right)\\ =total\left[Ca\right]-\left(0.0\text{12}\times \left[albumin\right]\right)+0.\text{479}\\ =total\left[Ca\right]+0.479-\left(0.012\;\left[albumin\right]\right)\\ =total\left[Ca\right]+0.012\left(0.479/0.012-\left[albumin\right]\right)\\ =total\left[Ca\right]+0.012\left(39.9\;-\left[albumin\right]\right) \end{array}$$

## Pre-publication history

The pre-publication history for this paper can be accessed here:



## Supplementary Material

Additional file 1 **Bland-Altman plot including 95% limits of agreement between adjusted calcium by the previously published equation and adjusted calcium by the locally derived equation (Note: mean difference = 0.09, 95% limits of agreement: 0.043 to 0.136, units = mmol/L).** Abbreviations: Adj. = Adjusted. Click here for file
