# Supplementary material for: Derivation and internal validation of an equation for albumin-adjusted calcium
Source: BMC Clin Pathol. 2008 Nov 27;8:12. doi: 10.1186/1472-6890-8-12 (PMC2607282; doi:10.1186/1472-6890-8-12)
Supplement: Additional file 1 — Bland-Altman plot including 95% limits of agreement between adjusted calcium by the previously published equation and adjusted calcium by the locally derived equation (Note: mean difference = 0.09, 95% limits of agreement: 0.043 to 0.136, units = mmol/L). Abbreviations: Adj. = Adjusted. [file 1472-6890-8-12-S1.doc]

**Supplementary Material** - Bland-Altman plot including 95% limits of agreement between adjusted calcium by the previously published equation and adjusted calcium by the locally derived equation (Note: mean difference=0.09, 95% limits of agreement: 0.043 to 0.136, units=mmol/L).

Abbreviations: Adj.=Adjusted
